# Using Google Trends to Determine Current, Past, and Future Trends in the Reptile Pet Trade

**DOI:** 10.3390/ani11030676

**Published:** 2021-03-03

**Authors:** Jose W. Valdez

**Affiliations:** German Centre for Integrative Biodiversity Research (iDiv) Halle-Jena-Leipzig, Puschstrasse 4, 04103 Leipzig, Germany; jose_w.valdez@idiv.de

**Keywords:** lizards, snakes, turtles, squamata, captive-bred, reptilia, gecko, serpentes, colubridae, bearded dragon, leopard gecko, crested gecko, tegu, ball python

## Abstract

**Simple Summary:**

Reptiles are one of the most popular exotic pets in the world. However, the most common pet reptiles are typically non-threatened, captive-bred, and are domestically obtained, which means they are largely unregulated and unmonitored, resulting in a large portion of the reptile pet trade remaining unknown. In this study, the most popular reptiles in the pet trade were examined using Google Trends and compared to the results from an online survey. The results determined that the most popular pet reptiles were by far bearded dragons, followed by ball pythons and leopard geckos. Conversely, green iguanas, Burmese pythons, chameleons, and red-eared sliders have declined the most in popularity. Meanwhile, crested geckos have been increasing in popularity more than any other reptile. The general finding of the study is that the reptiles declining in popularity were mostly wild-caught or restricted due to regulations, while current and future popular species were captive-bred and available in many varieties. The most popular species were also docile, medium-sized, easy to handle, and with relatively simple requirements. This study demonstrates that Google Trends can be a valuable tool in many research applications, particularly research topics which may otherwise be difficult to monitor and quantify.

**Abstract:**

Reptiles are one of the most popular exotic pets in the world, with over a third of all described species currently being traded. However, the most commonly available reptiles are typically non-threatened, captive-bred, and/or domestically obtained, which means they are also largely unregulated and unmonitored, resulting in a large portion of the reptile pet trade remaining unknown. In this study, the past, current, and future trends of the most popular reptiles in the pet trade were examined. Google Trends was used to determine the global popularity of the most popular pets from 2004 to 2020 and compared to the results from an online survey sent to individuals involved in the reptile trade. The most popular pets from the previous five years were also compared globally across regions and countries. The results determined that the most popular reptile species during the last decade is by far bearded dragons, followed by ball pythons and leopard geckos. Although the survey results were similar when asked what the top reptiles were, most respondents named ball pythons as the most popular reptile. However, when asked what reptiles had lost the most popularity during the previous decade, the survey respondents named green iguanas, Burmese pythons, chameleons, red-eared sliders, and green anoles, concurring with what was found with Google Trends. The reptiles thought to be more popular in the upcoming decade by the survey participants were blue-tongued skinks, tegus, uromastyx, crested geckos, and ball pythons—most of which did indeed show an increase in popularity during the last decade, as indicated with Google Trends. The results from Google Trends demonstrated that ball pythons and crested geckos have increased their popularity more than any other reptile in the last two decades. Reptile popularity also differed between countries, with bearded dragons the most popular reptile in Australia, Western Europe, the U.S., and Canada. Leopard geckos were the most popular reptile in Italy and Turkey, and ball pythons were the reptile of choice in Mexico, Indonesia, and India. The general finding of this study is that the reptiles declining in popularity were mostly wild-caught or restricted due to regulations, while current and future species were captive-bred and available in many varieties or morphs. The most popular species were also docile, medium-sized, and easy to handle, with relatively simple care requirements. This study demonstrates that Google Trends can be a useful tool for determining relative popularity among reptiles, or any other pet group, with results closely mirroring those obtained through direct surveying of people involved in the pet trade. However, unlike surveys, this analysis is quick, quantifiable, and can show what is popular and in-demand not only at the global level but at much finer scales. Thus, Google Trends can be a valuable tool in many research applications, especially in topics that may otherwise be difficult to monitor and quantify.

## 1. Introduction

Reptiles are one of the most popular exotic pets in the world, resulting in a quickly growing billion-dollar pet industry [1]. Their popularity as pets has made reptiles one of the most common animals in the wildlife trade [2], with evidence suggesting that over a third of all described reptile species are actively bought and sold around the world [3]. The largest reptile consumer markets are Europe and the U.S., with over 9 million reptiles being kept as pets in both regions [4,5,6]. In the U.S. alone, reptile ownership has more than doubled during the previous two decades, with an estimated 4.5 million homes currently containing at least one pet reptile [5,7]. This high demand for pet reptiles can lead to the overexploitation of many species, especially rare and hard-to-obtain species that are particularly attractive and can fetch high prices [3,8,9,10,11]. This has resulted in scientists becoming cautious to publish the locations of newly described species, as they are often heavily sought in the trade [8,12,13]. Yet, in some cases, previously undescribed species had already been well-established within the pet trade [14,15,16]. 

To protect vulnerable species from exploitation, the Convention on International Trade in Endangered Species (CITES) was enacted to regulate and monitor the international importing and exporting of wildlife, while organizations such as the U.S. Fish and Wildlife Service Law Enforcement Management Information System (LEMIS) monitor imports at the national level. Recent studies have used data from such agencies to help reveal the global trends and taxonomic extent of the international reptile trade, which often involves wild-caught, rare, and endangered species [2,3,17,18,19,20]. However, approximately 79% of traded reptile species are not subject to CITES trade regulations and are not systematically recorded, resulting in very limited data on most traded species [3,19,21]. Additionally, studies on the reptile trade do not typically distinguish between species destined for the pet trade or other purposes, such as the food or fashion industries, or even between wild-caught and captive-bred individuals [17]. Moreover, while thousands of species are globally traded, many illegally, the reptiles most commonly available to the average consumer are not going to be rare and expensive, but those that are more easily obtainable and widespread. Since these commonly traded species are typically not threatened, captive-bred, and/or are domestically obtained [3,20], they are also largely unregulated, resulting in a large portion of the reptile pet trade which remains unknown, untracked, and difficult to monitor. 

Trends within the reptile pet trade have also drastically shifted in recent decades, with an increase in domestic demand, a decrease in foreign supply, and overall less reliance on imported and wild-caught reptiles [1,18,19,20]. These trends can mostly be attributed to an increase in captive-breeding along with restrictive CITES regulations, which have helped to reduce the impact on many wild populations and lowered the proportion of CITES species within the trade [19,20]. This is highlighted by the U.S., which is both the largest importer and exporter of reptiles in the world and exemplifies the quick growth and success in captive-breeding, with over 10,000 hobbyists actively breeding reptiles. This has transformed the reptile pet industry from a small hobby to a viable profession worth over a billion dollars annually [1]. One study found that while the U.S. imported 900,000 live reptiles in 2009, it had exported more than 11 million [1,20]. The captive-breeding industry in the U.S. has been so successful that it is now the major exporter of species that are not even native to North America [1,20]. 

Although the reptile pet trade involves thousands of species, it tends to be dominated by a relatively small number of commonly traded and popular reptiles [3,20]. These species are typically captive-bred, inexpensive, charismatic, simple to set up, and include species appealing to consumers, such as bearded dragons, leopard geckos, ball pythons, corn snakes, and crested geckos. Collector demand has consequently shifted from rare, wild-caught species towards increasingly rare and expensive color and pattern variations, called morphs, of popular and easy-to-breed species [1]. The selective breeding of these “designer” reptiles can substantially increase the value of even the most common and unexciting species, which can be individually sold for up to tens of thousands of dollars [1]. However, whether they are normal color variations or morphs, these reptiles are commonly traded domestically from private breeders, expos, independent pet stores, and online retailers. Since these reptiles are domestically bred and traded, they remain largely unmonitored. Consequently, although representing a large segment of the reptile trade, relatively little is known in the scientific literature about these common and popular species. To fully grasp the extent and trends of the reptile trade, it is necessary to recognize these commonly traded species and help fill in the large knowledge gap in this growing trade.

An obvious method for investigating popular and commonly traded reptiles is through surveys. Surveying reptile hobbyists and traders has been used successfully to determine the number of households with pet reptiles [5], the extent of the illegal trade of some species [22], the knowledge and practices of husbandry by reptile pet owners [23,24,25], and the estimated mortality rates of pet reptiles [26]. However, surveys are difficult to obtain, time-consuming, and the individuals who respond are not always representative of the population of interest [27]. Obtaining survey results on the specific reptiles being sold and traded is a particular challenge since the individuals and organizations involved are especially circumspect to revealing information due to the ever-increasing threat of national and international legislation, as well as the perceived negative associations of the reptile trade with animal welfare, impacts on wild populations, human health, and the invasiveness of released species [28,29,30,31]. This became apparent during preliminary research for this study, after experts involved in the trade stated the difficulty of obtaining such information, with reptile breeders and even major pet stores unwilling to provide quantitative data or any information on the most common and popular reptile pets. 

An alternative method that avoids many of the issues of surveys is by analyzing the vast amount of data available on the internet. This methodology has already been used recently to examine the legal and illegal reptiles being traded online by scraping data from internet marketplaces [3,8,32]. However, while internet marketplaces have become a major component of the reptile trade, major websites such as Facebook have already banned the trading of animals, with many others following their example. Moreover, while a proportion of illegal and rare reptiles are obtained online [3], most reptiles are obtained from independent or chain pet stores [5]. Nevertheless, shopping habits and information-seeking habits have changed over the past 20 years, with internet search engines, particularly Google, now often the first place the public goes to for information. To infer public interest of reptiles, it may therefore be more conducive to examine information from search engines, as individuals would likely search for information prior to and after obtaining a reptile, including husbandry, sexing, health-related issues, tank requirements, etc. Luckily, internet search tools are a powerful source of information that can demonstrate explicitly reptile pet trends irrespective of where they are ultimately obtained. 

Google Trends has recently emerged as a useful tool for data-driven analysis of real-world phenomena by compiling data on search query volumes to determine the interest of certain topics over time. Using Google Trends to analyze spikes in search volumes can forecast economic indicators [33], financial markets [34], and even detect regional flu outbreaks a week before conventional monitoring systems [35]. Google Trends has also become an increasingly used and innovative tool in biodiversity and conservation, with the number of publications in the field growing over 50% per year [36]. It has already been used for examining trends in public interest on conservation issues [37,38,39,40], awareness of conservation projects [41], recognizing species bias in conservation resource allocation [42], the cultural aspects of environmental issues [43], and to track the timing of biological processes and geographic patterns of invasions [44]. Google Trends has also been shown to outperform survey-based indicators and can offer significant time and resource benefits for examining trends over time [45].

For this study, the current, past, and future trends of the most common and popular reptiles in the reptile pet trade were examined. First, an online survey was conducted and targeted to individuals involved in the reptile trade to determine what they believed to be the current, previous, and future popular reptiles. Google Trends was then used to establish the global popularity of reptile pets from 2004 to 2020 and compared to the survey results. To determine whether pet reptile trends differed among countries and regions, the five most popular pets and those predicted to be more popular in the future were also examined across countries with Google Trends. Since background information on the most common pet reptiles is lacking in the scientific literature, a general overview of the most important pet reptiles in this study is discussed.

## 2. Methodology

**Figure 1:**
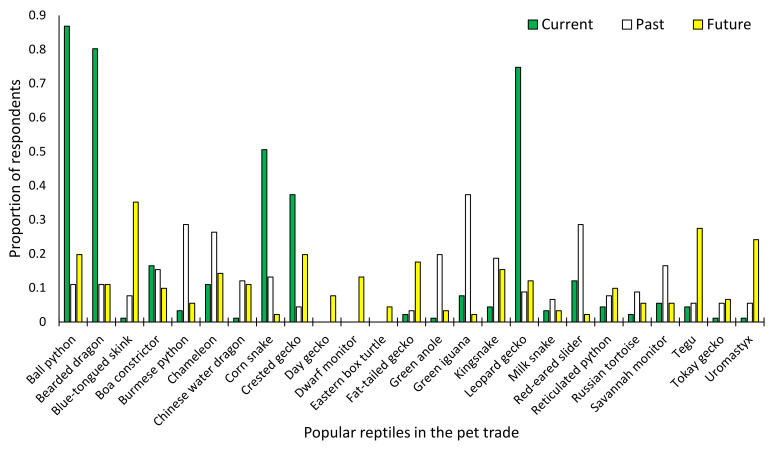
The proportion of respondents from an online survey indicating the top 3–5 reptiles they consider the most popular (Current), have lost much of their popularity in the previous decade (Previous), and will be much more popular in the upcoming decade (Future).

The most popular 25 pet reptiles were obtained by informally asking experts involved in the reptile trade, from online pet reptile websites and blogs, reviewing previous publications, and personal knowledge of the subject (Dataset S1; Figure 1). These reptiles were then included in a survey that was given to those involved in the reptile trade by targeting and posting on reptile pet groups on Facebook, ResearchGate, Twitter, Reddit, and personally contacting respective individuals. While many reptiles are typically represented by a single species (e.g., leopard geckos, ball pythons, crested geckos), other reptiles are known colloquially under a specific common name and may not always be distinguished between species (e.g., tegus, chameleons). To account for this, the survey questions asked participants to select reptiles from the list of the 25 reptiles along with the option of open-ended input so they could also include reptiles not listed. The survey included these questions:

1. What do you think are currently the 3–5 most popular reptiles in the pet trade?
2. What do you believe is the most popular reptile in the pet trade today?
3. What reptiles were popular in the last 10–15 years but not so popular today?
4. What reptiles do you think will be much more popular in the next 5–10 years then they are today?
5. How would you describe yourself?
6. Please state where you are from (optional)
7. What is your age? (optional)
8. How long have you been involved with the pet reptile trade? (optional) (Dataset S1).

To examine online search trends, Google Trends was used (freely available from www.trends.google.com, accessed on 5 January 2021). Google Trends data was obtained from a random and unbiased sample of Google search data which goes as far back as 2004, which is anonymized, categorized, and aggregated. Search index data is available at the country, state, and city levels. To measure search interest in a topic, values from Google Trends do not represent absolute search volume but reflect the number of searches for a particular term relative to the total number of Google searches. The values are normalized and indexed on a scale from 0–100, where 100 is the maximum search interest and each point is divided by that value [33]. A downward trend, therefore, does not necessarily indicate that the total number of searches for the term is decreasing, but that its relative popularity compared to other searches is decreasing. Only searches with a meaningful volume are tracked, so to increase accuracy it excludes searches made by very few people for a given time period or repeated searches from the same user over a short period of time [33]. 

In the survey, the reptiles most frequently cited as the most popular, having lost popularity, and predicted to be popular in the future were searched worldwide in Google Trends from 1 January 2004 to 31 December 2020. Five reptiles were compared at a time, since this is the limit of total search items that can be compared in Google Trends. However, to obtain comparable trends for all reptiles, the most popular reptile was chosen and subsequently compared to different groups of four other reptiles to obtain relative temporal trend values. This is important, since Google Trends is a measure of relative search patterns and different comparisons will produce different results, as it is highly sensitive to the benchmark term used [38,46]. To smooth out noise and visualize long-term trends from the samples, a yearly moving average was used. This also smoothed out the seasonal fluctuations, since reptiles were typically searched for more often in summer than in winter. Since words can have multiple meanings, *search topic* “Reptiles” was used. Using *search topic* improved the results by capturing the semantics of the search words and included all variations, such as misspellings, spelling variations, synonyms, plural or singular versions, different languages, and special characters. To further filter results to the correct context, the category of *pets* was chosen. The five current and future popular reptiles were also examined for the last five years and compared across countries, in the same way as described. To ensure equal comparisons, reptiles that typically comprise more than three species (e.g., chameleons) were excluded in this analysis. 

## 3. Results

**Figure 2:**
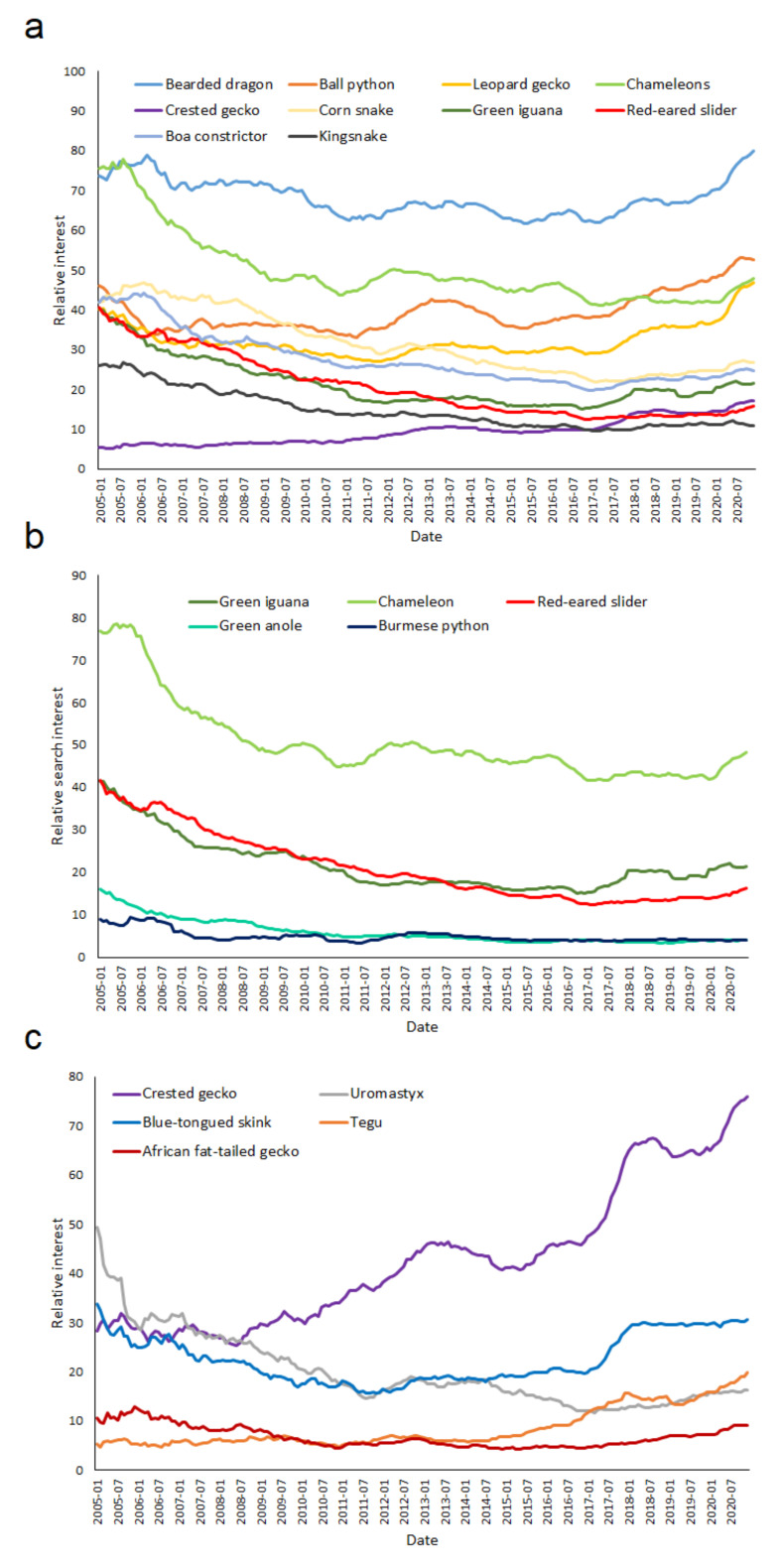
The (**a**) top ten most searched reptiles on Google, as well as the reptiles thought to (**b**) have lost most popularity in the previous decades and (**c**) will be much more popular in the next five to ten years, based on survey results. The numbers represent the yearly moving average search interest of reptiles relative to the highest point on the chart for the given date, with a value of 100 representing the peak popularity for a search term and a value of 50 indicating the term is half as popular.

A total of 91 participants took part in the online survey. Most were reptile hobbyists from the U.S., particularly Florida, followed by Germany, aged between 18 and 44, and involved in the pet trade from one to 20 years (Dataset S1). The reptiles that survey participants considered the most popular were ball pythons (86.8%), bearded dragons (80.2%), and leopard geckos (74.7%), followed by corn snakes (50.5%) and crested geckos (37.4%) (Figure 1). However, when asked specifically what they believed was the single most popular reptile, nearly half (46.2%) stated it was ball pythons, followed by a quarter for bearded dragons (22%), and leopard geckos coming in at third with 15.4% (Table 1). These results differed from those obtained from Google Trends, which demonstrated that bearded dragons were by far the most popular reptile, followed by ball pythons, leopard geckos, and corn snakes (Figure 2a). Chameleons, a group containing many species, was number eight in the survey as the single most popular (Table 1) but was the third most popular reptile in Google Trends, with a nearly identical score to leopard geckos (Table 1; Figure 2a). The other top ten reptiles in Google Trends were boa constrictors, green iguanas, crested geckos, red-eared sliders, and kingsnakes (Table 1; Figure 2a).

The reptiles labeled as having lost popularity during the last decade in the survey were green iguanas (37.4%), followed by Burmese pythons (28.6%), red-eared sliders (28.6%), chameleons (26.4%), and green anoles (19.8%) (Figure 1). These results were very similar to what was shown by Google Trends, with all the species showing a decline of around half or more from their highest popularity levels since 2004 (Figure 2b). However, the reptiles which had lost a considerable amount of popularity indicated by Google Trends were by far chameleons, followed by green iguanas and red-eared sliders (Figure 2a,b). Although these five species indicated by the survey have dropped in popularity since the early 2000s, most have remained relatively stable since the mid-2010s (Figure 2b).

The reptiles named in the survey as the most likely to show an increase in their popularity within the next decade were blue-tongued skinks (35.2%), tegus (27.5%), uromastyx (24.2%), crested geckos (19.8%), ball pythons (19.8%), and fat-tailed geckos (17.6%) (Figure 1). These results were also generally similar to what was found by Google Trends, with most of the species showing increases since 2004 (Figure 2c). Nevertheless, the general consensus was different when compared to Google Trends, with crested geckos showing by far the largest relative increase in popularity; nearly three times higher than in 2004. While blue-tongued skinks lost much of their popularity in the 2000s, they have become increasingly popular since the early 2010s. Tegus have also been steadily increasing in popularity during the last two decades. In contrast, the popularity of uromastyx has dropped by nearly four times from its highest levels (Figure 2c). However, when focusing just on the last three years, its popularity seems to remain steady (Figure 2c). Lastly, despite being the second most popular reptile and the most popular snake, the ball python has continued to increase in popularity since the 2010s (Figure 2a).

**Figure 3:**
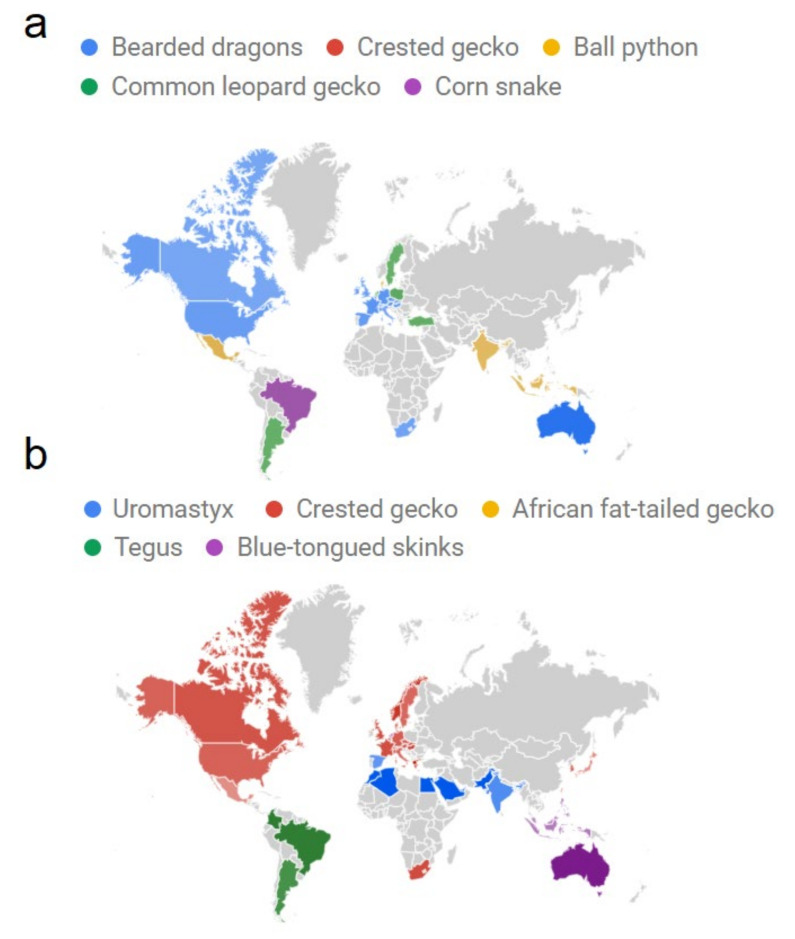
The reptile pets (**a**) most popular during the last five years and (**b**) growing in popularity, by country as indicated by Google Trends. Color intensity represents the percentage of searches relative to the reptiles compared.

When comparing the top five most popular reptile pets, countries differed in their preferences, with relative search percentages differing among these reptiles (Table S1). Bearded dragons were the most popular in Australia, with a total of 78% of searches compared to the other popular reptiles, the highest percentage of any reptile anywhere. Bearded dragons were also the most popular searched pet reptile in Western Europe, specifically France (46%), Germany (46%), Austria (45%), the Czech Republic (44%), Ireland (44%), and Spain (44%) (Figure 3; Table S1). The bearded dragon was also the most popular reptile in the U.S. (40%) and Canada (33%) (Figure 3a; Table S1). Leopard geckos were the most popular in Poland (42%), followed by Italy (36%), Turkey (36%), and Sweden (32%) (Figure 3a; Table S1). Ball pythons were the reptile of choice in Mexico (51%), Indonesia (44%), India (38%), Philippines (37%), and Malaysia (35%) (Figure 3a; Table S1). Meanwhile, corn snakes were the most popular reptile in Brazil (61%) (Figure 3a; Table S1). No country had crested geckos as the most popular reptile relative to the other currently popular reptiles (Table S1).

When looking at the reptiles thought to be more popular in the future, there were 32 countries with over 50% interest for a particular reptile (Table S1). This is in contrast to just three countries with over 50% search interest for a particular currently popular reptile (Table S1). Specifically, crested geckos were the most popular globally, especially in North America and Europe (Figure 3b; Table S1), comprising 50% or more of all searches relative to the other four reptiles. This reptile is especially popular in Hungary (72%), Norway (81%), and particularly Greece, with nearly all searches for crested geckos (Figure 3b; Table S1). Uromastyx were the most popular in Northern Africa and South Asia, particularly in Saudi Arabia, Qatar, Pakistan, Morocco, Kuwait, Egypt, and Algeria, where they comprise nearly all of their searches relative to the other four reptiles (Figure 3b; Table S1). Blue-tongued skinks were the most popular in Australia (94%), the Netherlands (44%), and Southeast Asia including the Philippines (44%), Indonesia (41%), and Malaysia (36%) (Figure 3b; Table S1). Although African fat-tailed geckos were not the most popular reptile anywhere, they had the highest percentage of searches in Taiwan (34%), second to crested geckos (40%) (Figure 3b; Table S1). 

## 4. Discussion

### 4.1. Current Reptile Pet Trends

The most popular reptile in the pet trade is the bearded dragon, which has maintained its popularity for over a decade. Although there are several species in the trade, all originating from the deserts of Australia, the most common is the central or inland bearded dragon (*Pogona vitticeps*). Despite the banning of the selling and exporting of Australian wildlife in the 1960s, the founder stock of today’s captive-bred populations was likely illegally smuggled out of Australia between 1974 and 1990 [47,48], with most of the captive population thought to have largely originated from several pairs imported from Germany in the 1980s [49]. This reptile has remained popular due to its appearance, personality, manageable size, and docile temperament. They are also the most attractive reptile for family and kids as they are relatively easy to handle, omnivorous, eat similar foods as humans, and relatively active during the day. Due to the relative ease and decades of selective breeding in captivity, there are now a wide variety of morphs available, which increases their appeal to both beginners and collectors alike. These results also coincide with a U.K. report that found bearded dragons were by far the most sold reptile in online classified ads [50]. While bearded dragons being the most popular reptile pet would not surprise anyone involved in the pet trade, this is nonetheless the first peer-reviewed study in the scientific literature demonstrating they are indeed the most popular pet reptile. 

The second most popular reptile, and by far the most popular snake, is the ball python (*Python regius*). While not nearly as popular in the early 2000s, it has substantially increased its popularity since the 2010s. The ball python is native to West and Central Africa and was one of the first reptiles to be selectively bred for morphs [1]. However, despite an increase in captive breeding, it is still typically cheaper to import them, and, unlike most of the other popular reptiles in the study, ball pythons are one of the most heavily traded CITES-listed species in the world [17,18]. This species was the second most globally traded reptile between 1996 and 2012 (after green iguanas), with nearly 3 million imported and accounting for 14% of total trade (with no other species accounting for more than 4%) [18,20]. Most of these wild-caught pythons originate from Togo, the world’s leading exporter of ball pythons. Between 2000 and 2010, Togo exported 80,000 pythons, representing over three-quarters of their reptile exports, and so many that CITES had to impose an export quota for reptiles originating from the country [18,19]. The species is likely popular as it is docile, low maintenance, relatively small (for a snake), easy to handle, and considered a good snake for beginners. Like the bearded dragon, this species has been selectively bred to enhance its appearance as well as its docility and affiliative behavior towards humans [29]. 

The third most popular single species reptile was the leopard gecko, or, specifically, the common leopard gecko (*Eublepharis macularius*). This ground-dwelling reptile comes from dry and semi-desert regions in Afghanistan, Iraq, Iran, Pakistan, and northwest India. It is thought to also be the first domesticated lizard in the reptile trade, with a long history of captive breeding resulting in a wide variety of morphs [47]. This species is nearly always captive bred and popular due to its hardiness, small size, and relative ease of care. However, they are likely not as popular as the bearded dragon since they are more fragile, nocturnal, and require live insects for sustenance. According to recent trends, the leopard gecko might soon pass the ball python and become the second most popular reptile in the trade. In this study, chameleons (Family Chamaeleonidae) were nearly identical and slightly ahead in popularity than the leopard gecko. Although chameleons are indeed a popular reptile, they may have had high search volumes simply because they consist of many species that are popular in the trade, specifically the Senegal chameleon (*Chamaeleo senegalensis*), veiled chameleon (*Chamaeleo calyptratus*), panther chameleon (*Furcifer pardalis*), and Jackson’s chameleon (*Trioceros jacksonii*). While many hobbyists distinguish between particular species of chameleons, the layperson usually does not; hence why they were clumped together as a group in the study. Nevertheless, the leopard gecko and chameleons were followed by the corn snake (*Pantherophis guttatus*) as the fifth most popular reptile. The corn snake is a small snake species from North America and is the second most popular snake after being overtaken in the 2010s by the ball python in popularity. However, they remain one of the most popular snakes as they are relatively small, have a calm temperament, easy to care for and tolerate being handled by their owners for long periods of time. For these reasons, they are seen as a good reptile for beginners and sold to first-time and intermediate consumers [1]. For both leopard geckos and corn snakes, there are a very large number of morphs available, with new variations available yearly, which also makes them attractive to collectors and increases their popularity in the trade.

Although the answers from survey participants on the most popular pets were somewhat similar to what was found in Google Trends, the particular conclusions differed. For example, surveyed individuals correctly named the top four single species reptiles, but when specifically asked what they believed to be the single most popular, nearly half named that it was the ball python. Since the ball python was the most popular snake, the reasons for this discrepancy may simply be that the participants who took the survey were hobbyists that just happened to be mainly involved with snakes. Within the reptile trade, individuals tend to focus their hobby on specific species or groups (snakes, lizards, geckos, turtles, etc.). Another reason may be that, as hobbyists in the trade, they may not be as enthusiastic about bearded dragons, which are more common in households. Since snakes appear more intimidating than lizards, most people may simply move on to ball pythons after they had their first encounter with a bearded dragon as a pet. Nevertheless, whatever the reason for this discrepancy, it demonstrates a known issue with surveys, in that no matter how much you attempt to obtain a representative sample, you cannot control who takes the survey, potentially leading to a biased outcome and incorrect conclusions.

### 4.2. Previous Reptile Pet Trends

When asked which reptiles used to be popular but lost their popularity in the previous decade, the results from the survey included green iguanas (*Iguana iguana*), Burmese pythons (*Python bivittatus*), red-eared sliders (*Trachemys scripta elegans*), and chameleons, which were validated by Google Trends. These are not surprising since they are all cited as the most globally traded CITES reptiles. In particular, the green iguana is the most traded reptile in the world, accounting for 46% of the total trade in the US from 1996 and 2012, with annual imports reaching one million in 1996 [18]. Although much of the population is captive bred [20], large numbers of iguanas are imported into the U.S. from Central America and then re-exported to Europe and Asia to supply the high demand for pets abroad [17]. However, the data very closely follows what was indicated by Google Trends, with the number of iguanas being traded decreasing by 62% between 2001 to 2012 [18]. One reason for a loss of popularity may be due to their large size and bad temperament, with many eventually being released into the wild, resulting in several non-native populations established across the U.S. [7]. 

Red-eared sliders are the most traded turtle species, with the U.S. exporting more than 100 million red-eared sliders between 1989 to 2009, comprising more than 80% of its total reptile exports [20,51]. Due to being easily obtainable, relatively low price, and small size as babies, they are a popular impulsive buy pet. However, most individuals die young due to a lack of appropriate care, and those that survive quickly outgrow their tank and require a much more elaborate setup as they are relatively dirty reptiles, with many owners eventually releasing them into the wild. These reasons, along with the large numbers being traded, is why red-eared sliders are now one of the most invasive reptiles in the world [20]. 

Another species that has lost popularity is the Burmese python (*Python molurus bivittatus*) from Southeast Asia, one of the largest snake species and the most exported large python [1]. This species started the morph trend in pet reptiles, when, in the 1980s, after National Geographic featured an article on the sale of a rare albino Burmese python, which resulted in American sellers eventually breeding and selling baby albino morphs for up to $2000 [1]. However, despite their docile temperament, they are very large, with the capacity to possibly kill their owners from severe bites or constriction. This, along with their large appetite and requirement for very large and secure enclosures, has led to many eventually being released into the wild and has now become established as an apex predator in South Florida and a major threat to native wildlife [52]. This has led to many restrictions in the state of Florida and other regions that have officially banned the Burmese python as a pet, which has more than likely contributed to their decline in popularity. 

Lastly, as previously stated, chameleons are very popular lizards mostly imported from African countries like Madagascar, Tanzania, and Togo [53]. Since the early 1980s, the U.S. has been the main importer of chameleons from Africa, accounting for 69% of their exports, but these trends have been declining after tougher regulations, partly to reduce the number of species being taken from the wild and after some chameleon species have become invasive in places like Florida [53]. Additionally, chameleons are known in the trade for rarely thriving in captivity due to their relatively difficult and specific husbandry requirements, health issues, and being stressed by handling, with many dying within a year. However, while Google Trends demonstrates their decline over time, they remain a relatively popular reptile in the U.S. This may be due to their very unique appearance as well as the ease of captive-breeding which has steadily increased to the point that the U.S. can fulfill its own demand, while also becoming an exporter of chameleons [53]. In the U.S., they are so popular, that, despite Florida having six invasive chameleon species caused by the pet trade, reptile hobbyists search for wild chameleons to bring them in as pets or to breed and sell them, with some selling for up to a thousand dollars [54]. 

### 4.3. Future Reptile Pet Trends

Looking towards the future of the trade, survey participants believed that blue-tongued skinks, tegus, uromastyx lizards, crested geckos, and ball pythons will increase in popularity within the next five to ten years. These results were also somewhat similar to what was indicated by Google Trends. However, compared to the other increasingly popular reptiles, by far the most popular of these was the crested gecko (*Correlophus ciliatus*), which has increased in popularity three-fold in the last two decades and was by far the most popular of this group, besides the ball python. This species undoubtedly has the most interesting history as it was thought to be extinct for well over a century until it was rediscovered in 1994 and has now become the second most common gecko in the trade [55,56]. Despite being protected, and exports prohibited under CITES, a few individuals were brought in by biologists in the mid-1990s from the Isle of Pines, with breeding lines eventually established in both the U.S. and Europe [56,57]. Due to the ease of breeding, which has quickly resulted in a variety of different color morphs, along with commercially available crested gecko food, their relatively small size, and capacity to thrive at room temperature without additional heating requirements, they are not only one of the top ten most popular lizards in the reptile trade but continue to steadily increase in popularity. 

While blue-tongued skinks lost some of their popularity in the 2000s, they have increased in popularity since the early 2010s. Blue-tongued skinks are represented by a few Australian species, but the most common species in the trade is the common blue-tongued skink (*Tiliqua scincoides*). This species is commonly captive bred and regarded as a great beginner pet due to their medium size, docility, hardiness, omnivore diet, and relatively simple requirements. However, as a non-CITES species with relatively low popularity compared to other species, little is known about this reptile in the pet trade. Meanwhile, the tegu has steadily increased in popularity in the last two decades and is the common name for several large species of lizards that belong to the family Teiidae and are native to Central and South America. The typical species in the pet trade are Argentine black and white tegu (*Salvator merianae*) and red tegu (*Salvator rufescens*). Although typically considered for more advanced reptile owners due to their large size and the large enclosures required, they are regarded as great pets due to their unusually high intelligence and ability to be housebroken, and are one of the only lizards that seem to enjoy human companionship. Lastly, the uromastyx is a genus of African and Asian agamid lizards, commonly called spiny-tailed lizards, and are friendly reptiles available in many different colors and varieties. However, their popularity has decreased significantly from a high in the 2000s, which may be due to their very low survival rate given the lack of understanding of their husbandry when they were initially taken from the wild. However, their popularity seems to have remained steady in the previous three years, which may be attributed to a better understanding by keepers of their dietary and environmental needs, with survival rates now surpassing those in the wild. Future studies will demonstrate whether these reptiles will indeed increase in popularity as suggested by survey participants. 

### 4.4. Global Patterns in the Reptile Pet Trade

Although the popularity of current and increasingly popular reptiles were generally similar around the world, especially regarding bearded dragons and crested geckos, it did differ between countries and regions. Bearded dragons were by far the most popular in Australia, with 78% of Google Trend searches compared to the other top four species, while blue-tongued skinks comprised 94% of searches for increasingly popular reptiles. This is likely due to Australia prohibiting non-native reptiles, with only native species currently allowed to be kept as pets. This means that, despite their global popularity, reptiles such as ball pythons, leopard geckos, crested geckos, and corn snakes cannot be kept as pets in the country. Bearded dragons were also the most popular searched reptile in the U.S., Canada, and Western Europe. Leopard geckos were the most popular in Italy, Turkey, Poland, Norway, and Vietnam, while ball pythons were the reptile of choice in Mexico, Indonesia, India, Netherlands, and South Africa. However, unlike Australia, with its heavily strong preference for bearded dragons, preferences were much more evenly distributed among the top three reptiles in other countries. Meanwhile, corn snakes were the most popular reptile only in Brazil and Finland. This follows other studies that have shown corn snake as the most popular pet reptile in Brazil [58]. For reptiles growing in popularity, crested geckos were similar to bearded dragons and very popular in North America and Europe. The other four reptiles rising in popularity were generally popular where they are native, such as tegus in South America and uromastyx in Africa. This likely explains why so many countries had more than 50% of searches for one particular reptile increasing in popularity. As with other aspects of the trade, the popularity of common reptiles across various countries were previously unknown in the scientific literature. 

### 4.5. Google Trends as a Research Tool

This study also demonstrates that Google Trends can be a quick and simple tool that can perform just as well as surveys but provide more accurate and less biased results. Also, unlike surveys, Google Trends is quick, quantifiable, and can show what is popular and in-demand not only at the global level but at much finer scales. Google Trends is especially valuable as it can be used to pinpoint trends not only in countries, but states, and even metropolitan areas of interest. Additionally, Google Trends provides a list of rising and related searches to the search topic, which can also be narrowed down geographically. This can be used to predict what is rising where and what other topics are being searched for in relation to the topic of interest. Since demand is particularly high for newly described species [8,12,13], Google Trends can be useful to determine what species are particularly in demand and where. This can be used for monitoring the trade and lead to more specific measures being taken to prevent the acquirement of banned species or target legislation for the pet trade across international and national boundaries.

#### 4.5.1. Future Directions

Google Trends can be used for much more than just demonstrating popular pet trends. For example, the reptile pet trade has contributed to the establishment of the most non-native species in the world [59], with establishment success driven by the number of release events [60]. This is especially concerning for popular and low-cost reptiles that are released when they become costly, have outgrown their enclosure, or just become inconvenient [61]. In the E.U. most of its non-native populations can be attributed to the release or escape of pets [62], with Florida alone having over 80 reptile species introduced via the pet trade [61,63,64,65]. Since non-native species can result in many negative ecological impacts, accurate predictions are necessary for implementing effective restrictions preventing their establishment. Since there can be a ten-year lag between when species become popular and when they are released and become established in the wild [7], using Google Trends can help predict and possibly prevent species from becoming invasive. Additionally, some of these common reptiles are riskier in terms of potential invasion to native ecosystems. Therefore, this data can be combined with predictive models to determine establishment potential [66,67], risk assessment [66,68], and potential to harm native ecosystems [66,68]. Additionally, climate matching parameters can be used to predict establishment success [69] and create spatially explicit maps to identify high-risk areas requiring preventative measures [68], which can be especially vital for climates more conducive for reptile establishment, such as the southern U.S., the neotropics, and southern Europe. These predictive models alongside Google Trends can not only save native fauna, but billions of dollars spent annually on the damage caused by their invasion [21]. 

Recognizing the most popular reptiles and identifying local trends can also benefit organizations such as animal welfare charities, animal rescues, and those associated with ecology and monitoring invasive and illegal species. This can be of particular value for rescue and rehoming charities, which can use these findings to anticipate which reptiles they may need to prepare for in the future. Additionally, it can be used to predict which species are most likely to be illegally traded. For example, a recent study found that nearly all reptile species smuggled into Australia were legal in the U.S., with an average of four years between when the species first appeared in the U.S. trade and when it was detected in Australia [70]. This data can even be of value for health organizations. For example, in Brazil, there were over 600 deaths from snakebites between 2015 to 2020, with the highest number from illegal venomous snakes which are often kept as pets [71]. Understanding which venomous species are actively being traded is important, especially since most countries will have little to low availability of anti-venom for non-native species. Lastly, understanding the popularity of species in other contexts can lead to important insights, with a recent study demonstrating that a species popularity was a larger driver of conservation translocations than its threatened status [72]. Understanding which species are popular can be useful for these organizations and agencies to more adequately target their educational resources and campaigns. 

#### 4.5.2. Limitations

Nevertheless, Google Trends does have its limitations. Many individuals may not Google their reptile-related questions but may ask within online forums, Facebook groups, or use other social media platforms. Some consumers may also never search online for their questions, but instead ask reptile traders in person. Since many popular pets are inexpensive, they may also be impulse purchases, which likely means customers have not completed an online search prior to their purchase and may also not search for information afterward. Google Trends may also not useful for rare and illegal reptiles which may be sold on the dark web or in person, and not likely to be searched on Google. Moreover, even if they are searched on Google, the data may not be available due to low search volumes. Conversely, a large number of searches may be due to some pet owners searching more about a particular reptile than others. However, since Google Trends eliminates repeated searches from the same user over short periods of time, this is not typically a concern. Additionally, some reptiles may not be searched for at all and taken directly from the wild from chance encounters, such as the cultural practice of non-commercial collection of threatened tortoises in Spain [73]. Nevertheless, this study shows that Google Trends can be used even for a quick and preliminary analysis of trends, which can be a starting point on where future efforts should be focused. 

### 4.6. Summary of Findings

This is the first study to quantitatively demonstrate the most popular species in the global reptile trade and those that have declined and are growing in popularity. Since many of these species are typically domestically bred, not threatened, and not listed in CITES, this part of the trade has remained largely unmonitored and unquantified, resulting in an incomplete understanding of the pet reptile trade [74,75]. The general findings of this study were that reptiles declining in popularity were mostly wild-caught or restricted due to regulations, while current and future reptiles were captive-bred and available in many morphs. The most popular species were docile, medium-sized, easy to handle, with relatively simple requirements, otherwise known in the trade as “beginner” reptiles. Nevertheless, while this study did not intend to be a comprehensive and definitive list of reptiles in the pet trade, it demonstrates that Google Trends can be a useful tool for determining the relative popularity of pet reptiles.

## 5. Conclusions

Reptiles have quickly grown into popular non-traditional pets, providing companionship and pleasure to their owners. While the global reptile trade causes significant threats to biodiversity due to the illegal overharvesting of wild-caught individuals, much of the reptile trade is now domestic and captive-bred. While this does not reduce the concern of the threat to native wildlife, understanding reptile trends can provide more comprehensive information about the reptile pet trade industry. Google Trends can be a useful tool for determining relative popularity among reptile pets, or other animal groups, with results closely mirroring those obtained through surveys. However, unlike surveys, this analysis is quick, quantifiable, and can show what is popular and in-demand not only at the global level but at finer scales. Google Trends can also be a valuable technology for many other applications, especially research areas that may otherwise be difficult to monitor or quantify.

## Figures and Tables

**Table 1:** The top ten most popular reptiles as indicated by an online survey and Google Trends. The Google Trend score represents the average relative monthly search interest for 2020.

Reptile	Survey Participants Naming It the Single Most Popular Reptile	Survey Rank	Google Trend Score	Google Trend Rank
Ball python	42	1	53	2
Bearded dragon	20	2	80	1
Leopard gecko	14	3	47	4
Corn snake	4	4	27	5
Crested gecko	3	5	17	8
Red-eared Slider	2	6	16	9
Reticulated python	2	6	7	11
Boa constrictor	1	8	25	6
Chameleon	1	8	48	3
Green iguana	1	8	21	7
Kingsnake	0	N/A	11	10

## Data Availability

The data presented in this study are available in Dataset S1 and Table S1.

## Supplementary Materials

The following are available online at https://www.mdpi.com/2076-2615/11/3/676/s1, Dataset S1: Survey results and Table S1: Percentage of total searches by country as indicated by Google Trends.
