# Supplementary material for: Using Google Trends to Determine Current, Past, and Future Trends in the Reptile Pet Trade
Source: Animals (Basel). 2021 Mar 3;11(3):676. doi: 10.3390/ani11030676 (PMC8001315; doi:10.3390/ani11030676)
Supplement: Supplementary file 1 [file animals-11-00676-s001.zip › Table S1-Percentage of total searches by country as indicated by Google Trends.docx]

**Table S1.** Percentage of total searches by country as indicated by Google Trends for a particular reptile relative to the other current and increasing popular reptiles, respectively. Search term percentage is relative to the total number of Google searches performed during the last five years in the specific country. Reptiles consisting of at least a third and half of all searches for the particular group within a specific country were represented by light and dark gray, respectively. N/A indicates there was not enough data for that group of reptiles due to low search volumes.

|  | **Currently popular reptiles** | | | | | **Reptiles increasing in popularity** | | | | |
| --- | --- | --- | --- | --- | --- | --- | --- | --- | --- | --- |
| **Country** | **Bearded dragon** | **Crested gecko** | **Ball python** | **Leopard gecko** | **Corn snake** | **Uromastyx** | **Crested gecko** | **African fat-tailed gecko** | **Tegu** | **Blue-tongued skink** |
| Algeria | N/A | N/A | N/A | N/A | N/A | >99% | <1% | <1% | <1% | <1% |
| Argentina | 28% | <1% | 28% | 31% | 13% | 29% | <1% | <1% | 71% | <1% |
| Australia | 78% | 1% | 7% | 5% | 9% | 1% | 4% | 1% | <1% | 94% |
| Austria | 45% | 3% | 19% | 16% | 17% | 31% | 57% | <1% | <1% | 12% |
| Belgium | 41% | 5% | 22% | 16% | 16% | 23% | 54% | <1% | <1% | 23% |
| Brazil | 14% | <1% | 12% | 13% | 61% | <1% | 1% | <1% | 98% | 1% |
| Canada | 33% | 11% | 25% | 19% | 12% | 18% | 63% | 4% | 2% | 13% |
| Colombia | N/A | N/A | N/A | N/A | N/A | <1% | <1% | <1% | >99% | <1% |
| Czechia | 44% | 4% | 9% | 23% | 20% | 21% | 55% | <1% | 3% | 21% |
| Denmark | 24% | 7% | 33% | 17% | 19% | 13% | 54% | <1% | 6% | 27% |
| Egypt | N/A | N/A | N/A | N/A | N/A | >99% | <1% | <1% | <1% | <1% |
| France | 46% | 5% | 19% | 16% | 14% | 14% | 67% | 2% | 8% | 9% |
| Germany | 46% | 2% | 15% | 15% | 22% | 24% | 58% | 2% | 4% | 12% |
| Greece | N/A | N/A | N/A | N/A | N/A | <1% | >99% | <1% | <1% | <1% |
| Hong Kong | N/A | N/A | N/A | N/A | N/A | <1% | 52% | 26% | <1% | 22% |
| Hungary | 36% | 3% | 17% | 22% | 22% | 28% | 72% | <1% | <1% | <1% |
| India | 25% | 3% | 38% | 13% | 21% | 55% | 18% | <1% | 6% | 21% |
| Indonesia | 17% | 1% | 44% | 27% | 11% | 19% | 16% | 5% | 19% | 41% |
| Ireland | 44% | 5% | 16% | 15% | 20% | N/A | N/A | N/A | N/A | N/A |
| Italy | 35% | 8% | 11% | 36% | 10% | 16% | 53% | 3% | 10% | 18% |
| Japan | N/A | N/A | N/A | N/A | N/A | 15% | 32% | 21% | <1% | 32% |
| Kuwait | N/A | N/A | N/A | N/A | N/A | >99% | <1% | <1% | <1% | <1% |
| Malaysia | 23% | 2% | 35% | 26% | 14% | 9% | 31% | 18% | 6% | 36% |
| Mexico | 11% | 1% | 51% | 30% | 7% | 29% | 30% | <1% | 20% | 21% |
| Morocco | N/A | N/A | N/A | N/A | N/A | >99% | <1% | <1% | <1% | <1% |
| Netherlands | 17% | 6% | 32% | 31% | 14% | 14% | 40% | <1% | 3% | 43% |
| Norway | 19% | 13% | 23% | 31% | 14% | 9% | 81% | <1% | <1% | 10% |
| Pakistan | N/A | N/A | N/A | N/A | N/A | >99% | <1% | <1% | <1% | <1% |
| Philippines | 24% | 1% | 37% | 31% | 7% | 24% | 20% | <1% | 12% | 44% |
| Poland | 7% | 14% | 27% | 42% | 10% | N/A | N/A | N/A | N/A | N/A |
| Qatar | N/A | N/A | N/A | N/A | N/A | >99% | <1% | <1% | <1% | <1% |
| Saudi Arabia | N/A | N/A | N/A | N/A | N/A | >99% | <1% | <1% | <1% | <1% |
| Slovakia | 40% | 7% | 15% | 21% | 17% | 14% | 68% | <1% | <1% | 18% |
| South Africa | 36% | 4% | 26% | 13% | 21% | <1% | 68% | 12% | <1% | 20% |
| South Korea | N/A | N/A | N/A | N/A | N/A | 19% | 55% | <1% | 7% | 19% |
| Spain | 44% | 3% | 20% | 23% | 10% | 46% | 33% | <1% | 8% | 13% |
| Sweden | 13% | 10% | 26% | 32% | 19% | 33% | 52% | <1% | 4% | 11% |
| Switzerland | 42% | 4% | 17% | 18% | 19% | 31% | 57% | <1% | 4% | 8% |
| Taiwan | N/A | N/A | N/A | N/A | N/A | 5% | 40% | 34% | <1% | 21% |
| Turkey | 28% | 8% | 15% | 36% | 13% | N/A | N/A | N/A | N/A | N/A |
| United Arab Emirates | N/A | N/A | N/A | N/A | N/A | 73% | <1% | <1% | <1% | 27% |
| United Kingdom | 40% | 7% | 18% | 15% | 20% | 11% | 66% | 4% | 2% | 17% |
| United States | 40% | 7% | 25% | 16% | 12% | 15% | 55% | 6% | 4% | 20% |
